# Factors associated with the SARS-CoV-2 infection among health professionals from university hospitals *

**DOI:** 10.1590/1518-8345.6482.3918

**Published:** 2023-05-12

**Authors:** Quézia Boeira da Cunha, Etiane de Oliveira Freitas, Daiane Dal Pai, José Luís Guedes dos Santos, Luciano Garcia Lourenção, Rosângela Marion da Silva, Tânia Solange Bosi de Souza Magnago, Fernanda Moura D’Almeida Miranda, Silviamar Camponogara

**Affiliations:** 1 Universidade Federal de Santa Maria, Hospital Universitário de Santa Maria, Santa Maria, RS, Brasil.; 2 Universidade Federal de Santa Maria, Departamento de Enfermagem, Santa Maria, RS, Brasil.; 3 Universidade Federal do Rio Grande do Sul, Escola de Enfermagem, Porto Alegre, RS, Brasil.; 4 Universidade Federal de Santa Catarina, Departamento de Enfermagem, Florianópolis, SC, Brasil.; 5 Universidade Federal do Rio Grande, Escola de Enfermagem, Rio Grande, RS, Brasil; 6 Universidade Federal do Paraná, Departamento de Enfermagem, Curitiba, PR, Brasil

**Keywords:** Coronavirus Infections, Health Personnel, Occupational Risks, Infection Control, Security Measures, Pandemics, Infecciones por Coronavirus, Personal de Salud, Riesgos Laborales, Control de Infecciones, Medidas de Seguridad, Pandemias, Infecções por Coronavírus, Pessoal de Saúde, Riscos Ocupacionais, Controle de Infecções, Medidas de Segurança, Pandemias

## Abstract

**Objective:**

to investigate factors associated with the SARS-CoV-2 infection among health professionals from university hospitals.

**Method:**

a multicenter, mixed approach study with concomitant incorporated strategy, carried out with 559 professionals in the quantitative stage, and 599 in the qualitative stage. Four data collection instruments were used, applied by means of an electronic form. The quantitative analysis was performed with descriptive and inferential statistics and the qualitative data were processed by means of content analysis.

**Results:**

the factors associated with the infection were as follows: performance of the RT-PCR test (p<0.001) and units offering care to COVID-19 patients (p=0.028). Having symptoms increased 5.63 times the prevalence of infection and adhering to social distancing most of the time in private life reduced it by 53.9%. The qualitative data evidenced difficulties faced by the professionals: scarcity and low quality of Personal Protective Equipment, work overload, physical distancing at work, inadequate processes and routines and lack of a mass screening and testing policy.

**Conclusion:**

the factors associated with the SARS-CoV-2 infection among health professionals were mostly related to occupational issues.

## Destacados

**(1)** Las cuestiones laborales influyeron en la protección de los profesionales durante la pandemia.

**(2)** La alta adhesión a las precauciones estándar y al distanciamiento no disminuyeron los casos positivos.

**(3)** La baja calidad de los EPP y las fallas en el triage dificultaron la protección en el trabajo.

**(4)** La infraestructura hospitalaria no favoreció el distanciamiento físico entre los equipos.

## Abstract (pt)

**Objetivo:**

investigar fatores associados à infecção por SARS-CoV-2 entre profissionais da saúde de hospitais universitários.

**Método:**

estudo multicêntrico, de abordagem mista com estratégia incorporada concomitante, realizado com 559 profissionais na etapa quantitativa, e 599 na etapa qualitativa. Foram utilizados quatro instrumentos de coleta de dados, aplicados via formulário eletrônico. A análise quantitativa foi realizada com estatística descritiva e inferencial e os dados qualitativos por meio de análise de conteúdo.

**Resultados:**

os fatores associados à infeção foram: realização de teste “RT-PCR” (p<0,001) e unidades com atendimento a pacientes com COVID-19 (p=0,028). Ter sintomas aumentou em 5,63 vezes a prevalência de infeção e aderir ao distanciamento social na maior parte do tempo na vida particular reduziu em 53,9%. Dados qualitativos evidenciaram dificuldades enfrentadas pelos profissionais: escassez e baixa qualidade de equipamentos de proteção individual, sobrecarga de trabalho, distanciamento físico no trabalho, processos e rotinas inadequadas e ausência de uma política de triagem e testagem em massa.

**Conclusão:**

os fatores associados à infecção por SARS-CoV-2 entre profissionais da saúde foram em sua maioria relacionados a questões ocupacionais.

**Highlights:**

**(1)** Occupational issues exerted an influence on the professionals’ protection during the pandemic.

**(2)** High adherence to standard precautions and distancing failed to reduce the number of positive cases.

**(3)** Low quality PPE and failures in screening hindered protection in the workplace.

**(4)** The hospitals’ infrastructure did not favor physical distancing between the teams.

## Introduction

With slightly more than two years of pandemic, the World Health Organization (WHO) confirmed the milestone of 500 million cases of the disease by the SARS-CoV-2 virus (COVID-19) and more than six million deaths, worldwide ^( 1)^ . Throughout this time, Brazil presented heterogeneous situations in relation to the SARS-CoV-2 infection, with disease acceleration and de-acceleration periods in the most diverse states and municipalities. In August 2022, it was the second country with the highest number of deaths recorded, totaling nearly 680,000, only behind the United States ^( 1)^ . 

In this health crisis context, the health systems played a fundamental role and health professionals faced extremely challenging work environments. Despite the strong feeling of ethical duty to work, health professionals endured concerns related to their own safety ^( 2- 3)^ . 

Occupational exposure is an important form of SARS-CoV-2 transmission and the hospital environment is considered as with high risk for contamination due to the hospitalization of patients infected with SARS-CoV-2, whether symptomatic or not ^( 4)^ . Control over spread of the virus among health professionals became fundamental, both because of the potential for lives lost and because of the sustainability of the health systems that, to a large extent, depend on the health of these workers. In addition to that, infected health professionals can become transmission vectors to other peers and to susceptible patients ^( 5)^ . 

From the beginning of the pandemic, the main protection measures recommended by the WHO involved hygiene care and social distancing, which started to be recommended at the global level. In relation to the health services, the use of standard precautions (SPs) stands out, which are measures that should be resorted to in the care provided to all patients, regardless of their diagnosis. SPs are measures historically adopted for the protection of health professionals against biological risk. During the pandemic, they were widely fostered as a strategy to prevent patient-professional transmission. Studies that analyzed the behavior of SARS-CoV-2 transmission in China at the beginning of the pandemic suggest that the adoption of protective measures combined with training and workload adequacy are effective in controlling SARS-CoV-2 transmission among health professionals ^( 4)^ . 

It is believed that researching factors associated with the SARS-CoV-2 infection among health professional is important to understand the impact of the disease on this population group. In addition to that, the identification of difficulties faced during this period can assist in devising future strategies to mitigate illness and death among health professionals in periods of similar health crises. The hypothesis according to this test is that unfavorable working conditions and protection measures are associated with SARS-CoV-2 infection among physicians and Nursing professionals. Given the above, the following questions emerge: (1) Which are the factors associated with the SARS-CoV-2 infection among physicians and Nursing professionals from university hospitals? (2) Which were the difficulties found in relation to the protective measures during the COVID-19 pandemic in university hospitals?

The main objective of the current study was the following: To investigate factors associated with the SARS-CoV-2 infection among health professionals from university hospitals. And, as secondary objectives, it sought to: assess adherence to the standard precautions and identify difficulties found in terms of the protective measures by health professionals during the pandemic.

## Method

### Study design

A multicenter study with a mixed approach and a concomitant QUANT (qual) incorporated strategy, conducted between September 2020 and October 2021. With this research strategy, it was sought to obtain analysis perspectives of the different types of data, contemplating the study objectives, considering the quantitative study as the main database of the research and the qualitative stage with secondary weight. In order to ensure methodological rigor of the study, the *Mixed Methods Appraisal Tool* (MMAT) ^( 6)^ was used and the internationally recognized Standards for Quality Improvement Reporting Excellence (SQUIRE) guide was followed to prepare the manuscript. 

### Setting

The scenario was comprised by five large-size university hospitals (with 151 to 500 beds), all reference for the treatment of COVID-19, located in the Brazilian South region, in the states of Rio Grande do Sul-RS, Santa Catarina-SC and Paraná-PR, four of them linked to the Brazilian Hospital Services Company ( *Empresa Brasileira de Serviços Hospitalares*, EBSERH). 

### Population

There were 19,491 health professionals (physicians, nurses, nursing technicians and assistants) working in these hospitals when data collection was initiated.

### Selection criteria

The quantitative stage, of the cross-sectional type, had the following participants as inclusion criteria: physicians and nursing professionals who worked in direct care to patients, at least since February 2020 (period when the epidemic began in Brazil). The workers excluded were those that were in the remote work modality, devoted to administrative work or in total distancing during the pandemic period.

In the qualitative stage, of the exploratory-descriptive type, in addition to the participants of the quantitative stage (physicians and nursing professionals), health professionals who worked as service managers, heads/coordinators of the units and professionals of the in-hospital infection control ( *Serviços de Controle de Infecção Hospitalar*, SCIH), workers’ health and permanent education services were also included. These criteria were informed to the participants when they were invited to take part in the research. 

### Definition of the sample

Convenience sampling was used to select the participants. All the workers with an email address registered at their institution were invited to take part in the study. Those who voluntarily agreed to fill in the data collection instruments comprised the final sample, totaling 559 professionals in the quantitative stage.

The sample of the qualitative stage was comprised by the physicians and health professionals who took part in the quantitative stage and answered the open questions included in the instrument (n=546). Health professionals working as managers or in infection control, workers’ health and permanent education services were also included, totaling 599 professionals. Thus, the qualitative data sample was closed due to saturation ^( 7)^ ; in other words, all the participants who answered the open questions were included in the study. 

### Data collection

Data collection took place online from September 2020 to October 2021 due to the sanitary restrictions in force during the pandemic. The invitations to participate in the research were made through email contacts, which included a brief presentation of the research and two electronic form links through the *Google Forms* ^®^ platform, one directed to physicians and nursing professionals and the other to managers and professionals from the In-Hospital Infection Control Center (IHICC), workers’ health and permanent education services. The target population was duly informed alongside each link. For the physicians and clinical Nursing professionals, the qualitative data questionnaire was available on the platform, immediately after the quantitative data collection instrument; however, the open questions were not mandatory. Monitoring reminders were sent every 15 days until collection was closed in each institution, which only happened after, at least, three collection attempts in each center. 

### Instruments used to collect the information

The data were collected by means of four instruments. The first contained sociodemographic data (age, sex, marital status, children) and occupational information (institution, sector, function, employment contract, predominant work shift and time of professional experience in years).

The second and third instruments were only applied to the physicians and clinical Nursing professionals (n=559). The second instrument, prepared by the researchers, consisted of 11 closed questions related to the COVID-19 pandemic and to protective measures. The third was the Standard Precautions Adherence scale, comprised by 13 items and validated for its use in Brazil ^( 8)^ . The scale is of the Likert type, its score varies from 1 (Always) to 5 (Never), its items are added up and a mean value is calculated, in order to provide a final score that varies between 1 and 5. The higher the mean value, the greater the adherence to the standard precautions. This scale was adapted in the writing of some items, in which the term “HIV” was substituted by “COVID-19”, in order to better contemplate the context of the current research. The adaptation was performed considering that there was no duly validated instrument available in Portuguese to measure adherence to the standard precautions during the pandemic. The adapted scale went through a content validation process with nine evaluators experienced in research studies in the workers’ health area. Both the items and the instrument as a whole were considered valid, with a Content Validity Index (CVI) ≥ 0.80, considered satisfactory ^( 7)^ . 

The fourth instrument was used to collect the qualitative data. For the physicians and clinical Nursing professionals, there was a questionnaire incorporated into the quantitative research protocol, with six open questions, prepared by the researchers and related to the care process for COVID-19 patients and adherence to standard precautions. For the managers and professionals of the infection control, workers’ health and permanent education services, the questionnaire contained four open questions, prepared by the researchers, which dealt with the process of organizing the care activities for COVID-19 patients and worker safety protocols used in the institution. It was answered by 546 care professionals and by 53 managers and professionals from the Infection Control, Workers’ Health and Permanent Education services.

It is noted that, before initiating data collection, a pilot test was carried out with eight Nursing professionals, who pointed out the need for a small change in the answer options of one of the closed questions from the Questionnaire of variables related to the COVID-19 pandemic. After this review, the instrument was sent to the study participants.

### Study variables

The dependent variable of this study was SARS-CoV-2 infection, defined by means of a previous positive test and reported in the second research instrument. The participants had three answer options: (1) I wasn’t tested; (2) Yes, I was tested and the result was positive and (3) Yes, I was tested and the result was negative.

The independent variables included in the analyses were as follows:

Sociodemographic and occupational variables: sex, marital status, children, institution, function, work sector, employment contracts, work shift and weekly hour load.

Variables related to COVID-19 pandemic (second instrument): care for COVID-19 patients in the service in which they work; provision of direct assistance to COVID-19 patients; having received guidelines/training; PPE use during the care provided to COVID-19 patients; manifestation of COVID-19 symptoms; performance of diagnostic tests, type and result; effective social distancing and belonging to a risk group for COVID-19.

Adherence to the standard precautions: this variable was measured with the Standard Precautions Adherence scale (third instrument). It is the mean of all 13 items that comprise the scale, where the higher the mean value, the greater the adherence to the standard precautions.

### Data treatment and analysis

The quantitative data were organized in an electronic spreadsheet in the form of a database, using Excel/Windows and analyzed in IBM-SPSS, version 25. The categorical variables were represented by their absolute and relative frequencies. The association of the categorical variables under study with the “SARS-CoV-2 infection” was performed by means of the chi-square test. The distribution of the quantitative variables was analyzed by means of the Shapiro-Wilk normality test. Due to non-normal distribution, they were represented by means of median and interquartile range and the Mann-Whitney test was used for the comparison with the “SARS-CoV-2 infection” variable. The comparison between the “Adherence to the SPs” and “SARS-CoV-2 infection” variables was performed by means of the Kruskal-Wallis test. The significance level adopted was 0.05. The Poisson regression model was also used to estimate adjusted and unadjusted Prevalence Ratios (PRs) and their respective 95% Confidence Intervals (CIs). The statistical significance of the prevalence ratios obtained in the Poisson regression models, with robust variance, was evaluated by means of the Wald’s test. The variables with significance values below 0.20 were included in the multiple model and a 5% significance level was adopted to maintain them in the final model, with “backward” selection of the variables.

The qualitative data were submitted to content analysis ^( 9)^ . The following stages were performed: 1) pre-analysis: organization of the dataset to be analyzed in order to render the initial ideas operational and systematize them; 2) exploration of the material: from the in-depth reading of the analysis material, seeking to establish categories and/or subcategories and 3) treatment of the results: it took place when the categories were worked on based on the authors of the literature review, adding to data interpretation by the researchers ^( 9)^ . The MAXQDA ^®^ software was used to assist in data organization, categorization and analysis. 

To ensure reliability of the qualitative data analysis, periodic meetings were held between the research team and members of the research group not involved in the survey to present the synthesis of the main findings obtained and discuss analytical possibilities. Through this discussion of peer analyses, it was possible to ensure consistency between the empirical data and the interpretations that were being constructed in the light of the researchers’ subjectivity and relevance for the research question and objectives ^( 9)^ . 

Integration of the quantitative and qualitative data aimed at complementarity of all the information, seeking explanations for the quantitative findings based on the analysis of the qualitative data. The interpretation stage was conducted by means of incorporation, after separately analyzing each of the data sources. The integration obtained was represented by means of an illustrative joint exposition diagram ^( 10)^ . 

### Ethical aspects

The Research Ethics Committees of all five participating institutions approved the study (approval numbers: 4,335,006; 4,466,661; 4,685,755; 4,348,898; and 4,501,805). All determinations set forth in Resolutions 466/2012 and 510/2016 of the National Health Council were met. To preserve the participants’ identity, their testimonies were identified by codes consisting in the letters “B”, “Ph”, “NT”, “NA” and “M” for nurses, physicians, nursing technicians, nursing assistants and managers and professionals from the infection control, workers’ health and permanent education services, followed by numbers associated with the order in which the questionnaire was received.

## Results

Table 1 shows the characterization of the participants in each of the study stages, verifying certain predominance of professionals belonging to the female gender and with a partner. In relation to work, most of the participants were Nursing professionals and had their employment contracts ruled by the Consolidation of Labor Laws ( *Consolidação das Leis do Trabalho*, CLT). 

**Table 1 -:** Sociodemographic and occupational characterization of the study participants. Paraná (PR), Santa Catarina (SC), Rio Grande do Sul (RS), Brazil, 2020-2021

	Quantitative stage (n=559)		Qualitative stage (n=599)			
	Care professionals (n=559)		Care professionals (n=546)		Managers, IHICC ^*^ , PE ^†^ , WH ^‡^ (n=53)	
Variables	N (%)	Md ^§^ [P25; P75]	N(%)	Md ^§^ [P25; P75]	N(%)	Md ^§^ [P25; P75]
**Age**		45 [39; 52.25]		45 [39; 45]		45 [39; 54]
**Sex**						
Female	432 (77.3)		424 (77.6)		38 (71.7)	
Male	127 (22.7)		122 (22.3)		15 (28.3)	
**Marital status**						
With a partner	444 (79.4)		437 (80.0)		41 (77.3)	
Without a partner	115 (20.6)		109 (20.0)		12 (22.7)	
**Children**						
Yes	407 (72.8)		410 (75.1)		19 (35.8)	
No	152 (27.2)		136 (24.9)		34 (64.2)	
**Profession**						
Nursing	397 (71)		391 (71.6)		36 (67.9)	
Medicine	162 (29)		155 (28.4)		13 (24.5)	
Pharmacy	-		-		3 (5.6)	
Physiotherapy	-		-		1 (1.8)	
**Employment contract**						
SLR ^||^	127 (22.7)		122 (22.3)		23 (43.4)	
CLT ^¶^	405 (72.5)		399 (73.1)		29 (54.7)	
Emergency	27 (4.8)		25 (4.6)		1 (1.8)	

* IHICC = In-Hospital Infection Control Center;

† PE = Permanent Education Service;

‡ WH = Workers’ Health Service;

§ Md = Median;

|| SLR = Single Legal Regime;

¶ CLT = *Consolidação das Leis do Trabalho* (Consolidation of Labor Laws)

A total of 132 (23.6%) professionals with positive test results for the SARS-CoV-2 infection were identified among the study participants during the research period. 54.6% of the participants had negative test results and another 21.8% had not still been tested or were waiting their results at the data collection moment.

Table 2 presents the sociodemographic and occupational variables by testing categories for the SARS-CoV-2 infection. Regarding the professional category, it was found that Nursing professionals had more positive tests for SARS-CoV-2 than physicians, although this difference was not statistically significant. 

**Table 2 -:** Descriptive analysis of the sociodemographic and occupational variables between the testing categories for the SARS-CoV-2 infection (n=437). Paraná (PR), Santa Catarina (SC), Rio Grande do Sul (RS), Brazil, 2020-2021

	SARS-CoV-2 infection			
	Total	Negative test result	Positive test result	p *
	437 (100)	305 (69.8%)	132 (30.2)	
	n (%)	n (%)	n (%)	
**Sex** ^†^				
Female	342 (78.3)	238 (54.5)	104 (23.8)	0.861
Male	95 (21.7)	67 (15.3)	28 (6.4)	
**Marital status** ^†^				
With a partner	347 (79.4)	245 (56.1)	102 (23.3)	0.468
Without a partner	90 (20.6)	60 (13.7)	30 (6.9)	
**Children** ^†^				
Yes	319 (73)	225 (51.5)	94 (21.5)	0.580
No	118 (27)	80 (18.3)	38 (8.7)	
**Sector** ^†^				
COVID area	45 (10.3)	30 (6.9)	15 (3.4)	0.630
Non-COVID area	392 (89.7)	275 (62.9)	117 (26.8)	
**Function** ^†^				
Nurses	326 (74.6)	221 (50.6)	105 (24)	0.118
Physicians	111 (25.4)	84 (19.2)	27 (6.2)	
**Predominant work shift** ^†^				
Day	289 (66.1)	207 (47.4)	82 (18.8)	0.244
Night	148 (33.9)	98 (22.4)	50 (11.4)	
**Age** ^‡^				
Median	43	43	43	0.568
P25; P75	37.5; 51	37.5; 51	37.25; 50	
**Time of professional experience (in years)** ‡				
Median	16	16	15	0.754
P25; P75	10; 24	10; 24	11; 24	

* p = Significance level (p<0.05);

† Chi-square test;

‡ Mann-Whitney test

In the bivariate analysis, the group of health professionals who tested positive for SARS-CoV-2 had a significant association with the following conditions: having undergone the Reverse Transcriptase Polymerase Chain Reaction (RT-PCR) test (p<0.001); having worked in units that offered care for COVID-19 patients, regardless of whether they were exclusive areas for COVID-19 or not (p=0.028); having had symptoms suggestive of COVID-19, that is, they were symptomatic (p<0.001); and having always been on social distancing as recommended by the WHO in other activities of their private life (p<0.001).

The association between adherence to SPs and SARS-CoV-2 infection was analyzed, not verifying any statistical difference between those infected and not infected (p=0.985).

Table 3 shows the Poisson regression model, single and multiple, considering a dichotomous outcome in relation to infection (positive and negative cases), with the negative cases as reference. 

**Table 3 -:** Estimated value of the prevalence ratio calculated by means of simple and multiple Poisson regressions (n=132). Paraná (PR), Santa Catarina (SC), Rio Grande do Sul (RS), Brazil, 2020-2021

	Simple regression		
	PRU ^*^	[CI ^†^ ]	p ^‡^
**Sex**			
Female	Ref ^§^		
Male	0.969	[0.683; 1.375]	0.861
**Marital status**			
With a partner	Ref ^§^		
Without a partner	1.134	[0.812; 1.585]	0.461
**Children**			
Yes	Ref ^§^		
No	1.093	[0.800; 1.493]	0.577
**Institution**			
Hospital A	Ref ^§^		
Hospital B	1.772	[1.110; 2.828]	**0.017**
Hospital C	1.533	[1.031; 2.279]	**0.035**
Hospital D	1.635	[0.998; 2.680]	0.051
Hospital E	1.728	[1.031; 2.897]	**0.038**
**Sector**			
COVID area	Ref ^§^		
Non-COVID area	0.895	[0.577; 1.391]	0.623
**Function**			
Nurses	Ref ^§^		
Physicians	0.755	[0.525; 1.087]	0.131
**Employment contract**			
SLR ^||^	Ref ^§^		
CLT ^¶^	1.393	[0.947; 2.050]	0.092
Emergency	1.576	[0.819; 3.033]	0.173
**Type of test**			
RT-PCR ^**^	Ref ^§^		
Serology	0.252	[0.098; 0.649]	**0.004**
Both	0.669	[0.467; 0.957]	**0.028**
**The service treats COVID-19 patients**			
No	Ref ^§^		
Yes	2.592	[1.128; 5.958]	**0.025**
**Guidelines and/or training in the institution about biosafety with a focus on preventing transmission of the new coronavirus**			
Yes	Ref ^§^		
No	1.222	[0.633; 2.360]	0.550
**Direct assistance provided to suspected or confirmed COVID-19 patients**			
No	Ref ^§^		
Yes	3.617	[0.955; 13.703]	0.059
Does not know	3.286	[0.561; 19.251]	0.873
**Symptoms suggestive of COVID-19**			
No	Ref ^§^		
Yes	6.141	[3.922; 9.615]	**<0.001**
**Social distancing in the private life activities**			
Always	Ref ^§^		
Most of the time	0.330	[0.230; 0.474]	**<0.001**
Occasionally	0.341	[0.094; 1.229]	0.100
Rarely	0.738	[0.236; 2.310]	0.602
**Risk group**			
Yes	Ref ^§^		
No	0.871	[0.641; 1.182]	0.375
	**Multiple regression**		
	PRA ^††^	[CI ^†^ ]	p ^[Table-fn t3f3b]‡^
**Symptoms suggestive of COVID-19**			
No	Ref ^§^		
Yes	5.634	[3.508; 9.050]	<0.0001
**Social distancing in the private life activities**			
Always	Ref ^§^		
Most of the time	0.461	[0.328; 0.647]	<0.0001
Occasionally	0.417	[0.131; 1.325]	0.138
Rarely	2.832	[0.834; 9.639]	0.095

* PRU = Unadjusted Prevalence Ratio (variables with significance below 0.20 were included in the multiple model);

† CI = Confidence interval (CI=95%);

‡ p = Significance level (p<0.05);

§ Ref = Reference;

|| SLR = Single Legal Regime;

¶ CLT = *Consolidação das Leis do Trabalho* (Consolidation of Labor Laws);

** RT-PCR = Reverse Transcriptase Polymerase Chain Reaction;

†† PRA = Adjusted Prevalence Ratio = “SARS-CoV-2 Infection” + “Institution” + “Employment contract” + “Direct assistance to suspected or confirmed patient” + “Type of test” + “The service treats COVID-19 patients” + “Symptoms suggestive of COVID-19” + “Social distancing in the private life activities”

The multiple model allows inferring that having symptoms increased by 5.63 times the prevalence of positive cases in relation to those who did not have symptoms, when adjusted for the variable related to social distancing. Those who stated being on social distancing most of the time had a 53.9% reduction in the prevalence of positive cases in relation to those who asserted that they always observe distancing in their private life, adjusted for symptoms suggestive of COVID-19.

The qualitative data contributed important subsidies to deepen understanding of these phenomena, as some difficulties faced for the protection of health professionals during the pandemic were identified in these hospitals.

Figura 1 presents qualitative data that contemplate the difficulties faced by health professionals in terms of the protective measures during the pandemic. In the first item, “Use of Personal Protective Equipment”, several issues related to use of these materials were identified. In item 2, “Work process, organization and routines”, frequent changes and non-uniformity in the guidelines were some of the difficulties identified. “Distancing in the work environment” was the third item and evidenced the obstacles to implement distancing in the hospitals. The fourth item, “Work overload”, shows conditions that led to an increase in the health professionals’ workload during the pandemic. In item 5, “Screening and testing policy”, some failures in relation to the screening of patients and professionals were evidenced in the institutions, in addition to difficulties in terms of COVID-19 testing. Figura 1 presents the participants’ testimonies representing each item that comprises the qualitative results. 

**Figure 1 -:** Difficulties found for the protection of health professionals during the COVID-19 pandemic. Paraná (PR), Santa Catarina (SC), Rio Grande do Sul (RS), Brazil, 2020-2021

**Difficulty identified**	**Use of Personal Protective Equipment**
Shortage and low quality of the equipment	*We’ve already been told to wear our fabric masks because there are no others. At this moment, the available masks are of awful quality, as an example, the elastic band keeps pulling the ears, leaving them red and in pain.* (NT33, Hospital A – Ward)
Prioritization of the COVID areas	*I understand that all professionals who are working in patient care (COVID or non-COVID), both in hospitalization as well as in the outpatient service, should receive an N95 mask, which was only provided to professionals in the COVID areas (emergency, ICU* ^*^ *, OMS* ^†^ *or COVID hospitalization).* (Ph100, Hospital C – Outpatient service)
**Difficulty identified**	**Work process, organization and routines**
Isolation of patients	*There’s lack of organization, in my work, on the part of the regulation, in hospitalizing suspected and positive COVID patients with other patients and it also puts us under risk.* (NT138, Hospital D – Ward)
Changes in the guidelines	*Mismatched information, different from institutional guidance. Frequent changes in guidelines and adequacy of the SOPs* ^‡^ . *Fear of contracting the disease reduces understanding of the guidelines.* (Ph44, Hospital C)
**Difficulty identified**	**Distancing in the work environment**
Co-living areas	*Despite the professionals’ technical qualification, it is necessary to make a weekly call to comply with safety course of action in the co-living areas. These places were the main sources for contamination among employees.* (Ph38, Hospital C)
Inadequate physical space	*It was very difficult at first and, to this day, we work in a small and overcrowded physical space.* (Ph12, Hospital D – Obstetric Center)
**Difficulty identified**	**Work overload**
Leaves and insufficient staffing	*Our greatest difficulty was the number of employees, due to leaves related to COVID-19. Temporary selection processes were opened and not enough people were hired to meet the need. This generated a tight schedule, work overload, stress and, consequently, problems in interpersonal relationships.* (M41, Hospital B)
**Difficulty identified**	**Screening and testing policy**
Screening and testing of the professionals	*The number of colleagues (from all sectors) who come to work with COVID-19 symptoms is shocking. I suggest a daily interrogation about symptoms before taking over the job.* (Ph30, Hospital C – CCRPA)
Screening and testing of patients, family members and companions	*The difficulties are in that the patients don’t have COVID symptoms and end up going to non-COVID wards and then they turn out to be positive and this exposes large groups of employees.* (M18, Hospital B) *As we’re Pediatrics, the family must be included in anamnesis, and there was a failure at this point, many times the family had COVID and this wasn’t taken into account, which exposed the other patients, because the child had no flu-like symptoms at that moment.* (M37, Hospital C)

* ICU = Intensive Care Unit;

† OMS = Occupational Medicine Service;

‡ SOPs = Standard Operating Procedures

**Figure 2 -:**
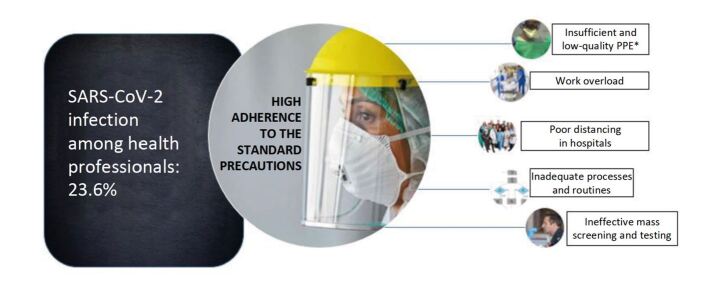
Diagram representing data incorporation: the complexity of protecting health professionals during the COVID-19 pandemic

The combination of the quantitative (QUANT) and qualitative (qual) approaches by means of incorporation allowed complementing interpretation of the findings, as shown in Figura 2. Although the use of protective measures, such as adherence to the standard precautions and social physical isolation, constituted an efficient and widely recommended barrier to contain the spread of SARS-CoV-2, the protection of the health professionals during the pandemic involved complex issues that go beyond the individual dimension. The incorporation of the qualitative data highlighted the importance of occupational issues such as infrastructure of the institutions, availability of protective materials and work processes. 

*PPE = Personal Protective Equipment

*PPE = Personal Protective Equipment 

## Discussion

In this study, factors associated with the SARS-CoV-2 infection were evaluated among health professionals from five university hospitals. Infection percentages of 16.7% and 26.4% were verified among physicians and Nursing professionals, respectively. These values are lower than those identified among health professionals (44.2%) in a university hospital from the city of Rio de Janeiro, one of the states most affected by the disease in Brazil ^( 11)^ . 

In the state of Ceará, a study conducted only with nurses identified 25% prevalence of COVID-19. In this same study, hospital nurses were 1.66 times more likely to have the infection than their Primary Care counterparts ^( 12)^ . 

In 13 European countries, the prevalence of SARS-CoV-2 among health professionals, between February and August 2020, showed strong heterogeneity with rates varying from 0.7% to 45.3% ^( 13)^ . Hospitals in Wuhan, China, reported infection rates of 3.5% to 29% among health workers at the beginning of the disease outbreak, when the protective measures were still inconsistent ^( 14)^ . 

It was identified that Nursing professionals had more positive SARS-CoV-2 cases when compared to physicians. Official Ministry of Health data from August 2020 pointed to Nursing as the category most affected by COVID-19 in Brazil, with 88,358 (34.4%) infection cases among nursing technicians/assistants, 37,366 (14.5%) among nurses and 27,423 (10.7%) among physicians ^( 15)^ . 

Studies conducted in several countries have shown that Nursing professionals were the most infected ^( 16- 17)^ . Among the possible reasons for the high number of contaminated Nursing professionals, closer and longer contact with patients stands out, involving activities performed at the bedside such as drug administration and also the performance of higher risk procedures, such as aspiration of tracheal secretions, in addition to being the first response line in case of complications in the patients ^( 16)^ . 

It was also observed that the type of test associated with the infections was RT-PCR. The RT-PCR method (which detects the virus) has been approved by the WHO as the “gold standard” for diagnosis and detection of the disease. However, immune response tests are also important for determining protective immunity in several infected population categories ^( 11)^ . 

As for the symptoms, it was verified that most of the professionals (86%) who tested positive were symptomatic and having symptoms increased the prevalence of positive cases by 5.63 times. Even so, it is necessary to consider that 14% of the infection cases in this research were asymptomatic. In an analysis based on 15 studies, the researchers identified 40% pooled prevalence of health professionals infected by COVID-19, diagnosed with RT-PCR, who had no symptoms at the time of diagnosis ^( 16)^ . Although asymptomatic transmission is still controversial, the potential for silent transmission is still an issue that needs to be addressed efficiently. 

Although a higher infection rate was not identified in professionals who worked in the exclusive areas for coping with COVID-19, those who tested positive for SARS-CoV-2 were active in units that offered care for cases of COVID-19 positive patients. This suggests that professionals in areas not exclusive to COVID-19 were also exposed to the infection and at greater risk due to the lower availability of Personal Protective Equipment (PPE) in these loci or to the lower adherence to use of these items and other protective measures. This hypothesis is corroborated by a systematic review of 46 studies which showed that most of the professionals positive for SARS-CoV-2, using RT-PCR, worked in hospital wards, followed by operating rooms and surgical services ^( 16)^ . A study carried out at a university hospital in Verona, Italy, identified that almost two-thirds of the health professionals with anti-SARS-CoV-2 seroprevalence were workers with a history of previous close contact with a COVID-19 case, in the hospital ^( 18)^ . 

In relation to the origin of the infection, few studies have analyzed the potential source of SARS-CoV-2 transmission among health professionals. Although the literature evidences a higher prevalence of infection in this population group when compared to data from the general population, the possibility of evaluating the impact of in-hospital infection *versus* the one acquired in the community is still limited. The results of a systematic review suggest that household contacts can play a significant role in infection, especially due to the rapid circulation of the virus in the community. In addition to that, the infection of asymptomatic carriers might exert an influence in view of the high number of professionals identified with this condition ^( 16)^ . 

In the current study, the difficulty maintaining distancing between professionals in the work environment during the pandemic was one of the main obstacles to protection pointed out by the managers, which may play an important role in transmission of the virus among these individuals. The professionals’ co-living environments, such as cafeterias and rest areas were perceived as the most critical for virus transmission, partly due to relaxation of the protective measures in these places, but also due to the small physical space that favors crowding.

A number of authors point out that awareness raising strategies for changing routines and habits are highly relevant, even during meals and group meetings ^( 4)^ . In addition to that, the importance of distancing in potentially neglected situations, such as in elevators, public transportation means (buses or vans) and clinical meetings, need to be considered ^( 19)^ . In university hospitals, maintaining this distancing can be especially challenging, considering the higher number of people involved in care and the need for interaction between residents and preceptors to discuss clinical cases, a situation that makes other control measures even more prevalent. 

One of the results found in this research is that the professionals who reported adopting social distancing in their private life activities most of the time (between 50% and 95% of the time) had a 53.9% reduction in the prevalence rate for the COVID-19 infection, when compared to those who stated having always observed distancing (more than 95% of the time). This finding allows for two interpretations. The first is that, even performing effective social isolation in environments outside of work, health professionals are doubly exposed to the COVID-19 infection due to close contact with patients and contact with colleagues, in environments that often do not favor distancing.

Another possible interpretation permeates the issues related to the workers’ mental health during the pandemic. It is known that, despite being a measure strongly recommended by the WHO, isolation brought about an invisible cost related to emotional problems, which can lead to greater susceptibility in individuals, especially for those who need to work directly with infected people and suffer more from anxiety, which might reduce the ability to understand the guidelines.

Several research studies have analyzed workers’ mental health during the pandemic. In this sense, exhaustion, anxiety, depression and fear levels were identified among nurses from American health services, with frequent changes in policies and procedures as main stressors, in addition to lack of PPE items and other supplies necessary for protection ^( 3)^ . In Canada, intensive care nurses from a university hospital also reported psychological distress related to frequent changes in policies and information related to infection control and PPE items, which occurred in response to new information on coronavirus transmission. The updates to the guidelines often conflicted with the previous ones and/or across the different sources (departments, management areas and governmental spheres), generating frustration in the professionals, already overwhelmed with patient care and who felt unable to stay updated and without knowing what information to follow and what the best practice was ^( 20)^ . 

In this study, lack of PPE items in adequate numbers and quality for the professionals’ protection was found in countless reports. In relation to the type of mask to be used, the Brazilian Ministry of Health recommended surgical masks for direct assistance to patients and the use of an N95/PFF2 mask or equivalent for the care of suspected or confirmed COVID-19 patients, during the performance of potentially aerosol-generating procedures ^( 21)^ . That recommendation justifies the decision to provide N95/PFF2 masks only to COVID-19 areas, adopted at the beginning of the pandemic by the institutions that participated in this research. However, a study conducted in China with 493 health professionals showed that the infection risk of a group that used surgical masks was significantly higher when compared to the group that used N95 masks (OR = 464.82, 95% CI: 97.73, ∞), although this latter group had a significantly higher exposure to infected patients ^( 14)^ . 

Consequently, although it was an official recommendation, the decision to provide more effective masks only to areas intended for the treatment of COVID-19 or for confirmed cases, may have contributed to the exposure of several health professionals in the care of patients who, at first, did not pose any contamination risk. On the other hand, it is necessary to consider that focusing only on mask use can produce a false sense of safety, which may increase dissemination of the virus if it is not accompanied by more fundamental measures for infection control, such as hand and environmental hygiene and use of other PPE items ^( 19)^ . 

The lack of PPE items, in times of overload of health systems, was an important factor related to infection among health professionals, according to research studies from the initial period of the pandemic carried out in China, Italy, Spain and the United States ^( 4)^ . In the Brazilian context, there is diverse evidence that the deficit of PPE items predates the crisis situation, and it is predictable that, in global disasters, the country would go through periods of scarcity and shortage. In this sense, ensuring safe conditions for the professional practice, with adequate physical barriers provided by PPE items, is a *sine qua non* condition for the work activity, which cannot be relaxed or improvised under any circumstances ^( 5)^ . 

This study identified that work overload is one of the difficulties for the professionals’ safety. During a pandemic, it is common for health professionals to work for many hours, without breaks and under significant pressure, increasing occupational exposure to the infectious agent and exposing workers to diseases and accidents. Thus, it is essential that the professionals have adequate and sufficient rest time to recover from physical and psychological wear out ^( 5)^ . 

In the participants’ reports, it was identified that suspected and confirmed COVID-19 patients were hospitalized in the same place as patients with other pathologies. These reports were mainly verified in ward areas and obstetric centers and possibly reflect the period of highest overcrowding in the institutions, when the beds devoted in areas for the exclusive care of COVID-19 patients were fully occupied.

Given the aforementioned, it is considered that spread of the infection within the institutions could be minimized in view of some measures described in the literature, such as: early implementation of contact and droplet precautions for all symptomatic patients and, in case of doubt, err by excess; daily reassessment of all patients hospitalized due to COVID-19 symptoms, considering cases in which the infection was in the incubation period at admission, or even in the case of exposure to the virus in the hospital environment itself; and adoption of a low threshold to test patients with mild symptoms, favoring early identification of the positive cases ^( 19)^ . 

Other strategies that are considered important to control transmission in the hospital environment include the following: isolating cases of symptomatic professionals, testing them frequently, clear and easy communication, and simple and accessible protocols ^( 4)^ . In Italy, a country heavily affected by the pandemic, Physicians recommended screening health professionals at the beginning of the work shift and rapid testing of all those that presented any symptoms suggestive of the disease (even if mild or without fever) and also for contacts of suspected or confirmed cases ^( 22)^ . 

Testing of health professionals in pandemic situations is an important tool for health care maintenance, as it provides early symptomatic treatment, enabling a shorter return to work, reducing absenteeism ^( 23)^ . In this sense, the Brazilian Ministry of Health recommended that health services implement non-punitive policies, allowing professionals with respiratory infection symptoms to be distanced from work to undergo home isolation ^( 21)^ , a measure that was adopted by the institutions that participated in this research. 

The WHO recommended testing all health workers, even without symptoms, as one of the strategies to contain infection among such workers ^( 24)^ . However, it was observed that a high percentage (21.8%) of the professionals who participated in the study had not been tested and/or were awaiting their results. 

Problems in screening and performing tests among patients and professionals were also verified in a research study that sought to analyze the work environment of nurses from Brazilian university hospitals ^( 25)^ . A study carried out at the national level between April and June 2020 identified that only 27% of the health professionals had undergone some type of testing for COVID-19 ^( 23)^ . This situation probably resulted from the operational limitations related to the supply of tests, considering the global shortage of inputs during a given period of the pandemic and due to slowness in processing the analyses ^( 5)^ . In this sense, the difficulty of testing, especially in groups more vulnerable to infection, such as health professionals, constituted a significant barrier that prevented dimensioning the actual magnitude of the pandemic. 

As limitations of this study, we can point to its cross-sectional design, which makes it difficult to establish cause and effect relationships. In addition to that, the long data collection period can also be considered a limitation, especially given the rapid changes in the epidemiological scenario, in addition to the vaccination that was initiated during the study.

Despite this, the study has important implications for the formulation of public policies and for the management of health services aiming at better resource planning in order to reduce transmission of SARS-CoV-2 and other infectious diseases in hospitals. Thus, it is possible to mention the following strategies: provision of adequate and sufficient PPE items to all the professionals providing direct assistance to patients or subjected to the biological risks generated by them; adequate staffing, with a technical safety index; improvements in information and processes involving the professionals’ safety, with clear assistance protocols accessible to all and investment in appropriate work environments, with healthy places for meals, rest, meetings, among others.

In addition, the research brings about contributions to the health area from a scientific perspective, considering the use of a mixed research design, which is an emerging methodological approach with the potential to expand the scope of knowledge construction in the area. It is also possible to point out some directions for future research, such as the development of research studies seeking to intervene in the work environment, seeking permanent improvements with regard to workers’ health in hospitals.

## Conclusion

Despite the high adherence to the standard precautions and social distancing recommended by the WHO, the percentage of health professionals with positive tests for SARS-CoV-2 was high. Most of the positive cases were symptomatic. The findings evidenced that the protection of such professionals was hampered by occupational issues, such as scarcity and low quality of Personal Protective Equipment, work overload, difficulty performing physical distancing in the workplace, inadequate work processes and routines, and absence of a more effective mass screening and testing policy.
